# Approximate series solution of multi-dimensional, time fractional-order (heat-like) diffusion equations using FRDTM

**DOI:** 10.1098/rsos.140511

**Published:** 2015-04-29

**Authors:** Brajesh K. Singh, Vineet K. Srivastava

**Affiliations:** 1 Department of Applied Mathematics, Babasaheb Bhimrao Ambedkar University, Lucknow 226025, Uttar Pradesh, India; 2 ISRO Telemetry, Tracking and Command Network (ISTRAC), Bangalore, Karnataka 560058, India

**Keywords:** multi-dimensional diffusion equation, Caputo time-fractional derivative, Mittag–Leffler function, fractional-order reduced differential transform method, exact solution

## Abstract

The main goal of this paper is to present a new approximate series solution of the multi-dimensional (heat-like) diffusion equation with time-fractional derivative in Caputo form using a semi-analytical approach: fractional-order reduced differential transform method (FRDTM). The efficiency of FRDTM is confirmed by considering four test problems of the multi-dimensional time fractional-order diffusion equation. FRDTM is a very efficient, effective and powerful mathematical tool which provides exact or very close approximate solutions for a wide range of real-world problems arising in engineering and natural sciences, modelled in terms of differential equations.

## 2. Introduction

The history of fractional calculus is very long and the first idea appeared in Leibniz's letter in 1695. In the beginning, for up to three centuries, fractional calculus theory was restricted to only pure mathematics. Later on, fractional partial differential equations began receiving great attention among researchers due to their tremendous applications in the fields of physics, chemistry, ecology, biology and engineering [1–11]. It has been found that derivatives of non-integer order are very effective for the description of many physical phenomena such as rheology, damping laws and diffusion process. These findings have invoked the growing interest in studies of fractional calculus in many branches of science and engineering.

This work is concerned with the time fractional multi-dimensional diffusion equation:

2.1 $$D_{t}^{\alpha }u=\nabla \cdot (D(u,r)\nabla u),\;0<\alpha \leq 1,$$

subject to the initial condition

2.2 $$u(r,0)=u_{0}(r),\;r\in R^{3},$$

where $D_{t}^{\alpha }u=\partial^{\alpha }u/\partial t^{\alpha }$ denotes Caputo fractional derivative of *u* of the order *α*, *u*(*r*,*t*) denotes the density of the diffusing material at the point *r*=(*x*,*y*,*z*) and time *t*, and *D*(*u*,*r*) denotes the diffusion coefficient for *u* at the point *r*. If the diffusion coefficient is independent of the density (i.e. *D*(*u*,*r*)=*σ*^2^ is a constant), then equation (2.1) reduces to the fractional order multi-dimensional heat equation, i.e. $D_{t}^{\alpha }u=\sigma^{2}\nabla^{2}u$, which represents the distribution of heat in a given domain. In particular, for *α*=1 and for the constant diffusion coefficient, equation (2.1) becomes the classical multi-dimensional diffusion equation, *u*_*t*_=*σ*^2^∇^2^*u*, which has received numerous applications in a wide variety of linear and nonlinear systems in physics, chemistry, ecology, biology and engineering. To describe processes exhibiting diffusive-like behaviour, it is also applicable, for example, to diffusion of alleles in a population in population genetics. The fractional order diffusion equation (2.1) has been applied in modelling to describe practical sub-diffusive problems in fluid flow process and finance [12]. In the one-dimensional case, the fundamental solution was computed for the first time in 1996 [13], later for the multi-dimensional case [14] and recently in a simpler form [15].

In the literature, various analytical and numeric approaches have been developed for the solution of such types of fractional-order PDEs. The numerical schemes are a finite difference scheme with non-uniform time steps [16–18], a higher order numerical scheme [19], an implicit finite-difference scheme [20],a compact difference scheme [21],a composite scheme combining alternating directions implicit approach with Crank–Nicolson discretization and Richardson extrapolation [22]. Before 1998, there was no analytical approach to solve such types of equations. In 1998, variational iteration method (VIM) was proposed to solve fractional differential equations [23–25]. Using this idea, several articles [25–34] have been written for more complex fractional differential equations showing the effectiveness and accuracy of the method. For more schemes, one can refer to recent contributions by Mustapha and colleagues ([35–37] and references therein).

In 2002, the Adomian decomposition method (ADM) was suggested by Shawagfeh [38] to solve fractional differential equations. However, in ADM, it was found to be very difficult to compute the Adomian polynomials [39–45]. As an alternative approach, He [46] proposed the homotopy perturbation method (HPM) for solving such differential equations. Momani & Odibat [47–49] used HPM to solve various fractional PDEs. Analytic solution of fractional diffusion as well as wave equations has been obtained by Jafari & Momani [50] using modified HPM, Das [51] used VIM, and ADM was applied by Ray & Bera [52]. Fractional heat and wave-like equations with variable coefficients have been solved using the homotopy analysis method by Xu & Cang [53], and also by VIM [54].

Recently, the fractional order multi-dimensional diffusion equation was solved using a modified homotopy perturbation method (M-HPM) by Kumar *et al.* [55]. The method was based on Sumudu transform [55] and HPM. In this method, homotopy parameter *p* is introduced to expand the solution in series form, whereas the nonlinear term is expanded by using He's polynomial [46–49]. But researchers found that it is a very difficult task to calculate He's polynomial, and the major drawback of these approaches is their complicated and huge calculations. The fractional reduced differential transform method (FRDTM) has been developed by Keskin & Oturanc [56] in order to overcome such type of drawbacks. FRDTM is the most easily implemented analytical scheme for the exact solution of both linear and nonlinear fractional differential equations. It is a very effective, reliable, efficient and very powerful analytical approach [57–60].

In this paper, we present an approximate analytical solution of the time fractional multi-dimensional (heat-like) diffusion equation of the order *α*(0<*α*≤1) in a series form which converges to exact solution rapidly, using FRDTM. The rest of the paper is organized as follows: basic preliminaries and notations on fractional calculus theory are revisited in §3. The preliminary on FRDTM is given in §3*a*. In §4, exact solutions of four test problems of time fractional-order multi-dimensional diffusion (heat-like) equations are obtained, while §5 concludes the study.

## 3. Basic definitions and notations on fractional calculus theory

Several definitions of fractional integrals or derivatives are available in the literature, given by Riemann–Liouville, Gruunwald–Letnikow, Caputo, etc. Here, we revisit only the basic definitions and preliminaries based on fractional derivatives and fractional integrals, which we will use to complete our study. Following are the most reasonable and meaningful definitions due to Liouville [4,11]:

**Definition 3.1 [4,11]**

Let μ∈R and m∈N. A real valued function $f:R^{+}\to R$ belongs to $C_{\mu }$ if there exists k∈R, *k*>*μ* and g∈C[0,∞) such that *f*(*x*)=*x*^*k*^*g*(*x*), for all $x\in R^{+}$. Moreover, $f\in C_{\mu }^{m}$ if $f^{\left(m\right)}\in C_{\mu }$.

**Definition 3.2 [4,11]**

The Riemann–Liouville fractional integral of $f\in C_{\mu }$ of the order *α*≥0 is defined as

3.1 $$J_{t}^{\alpha }f(t)=\left\{\begin{array}{ll} f(t) & \text{if }\alpha =0,\\ \frac{1}{\Gamma (\alpha )}\int_{0}^{t}{\left(t-\tau \right)}^{\alpha -1}f(\tau )\;d\tau , & \text{if }\alpha >0, \end{array}\right.$$

where *Γ* denotes gamma function: $\Gamma (z)=\int_{0}^{\infty }e^{-t}t^{z-1}\;dt,z\in C$.

In their work, Caputo & Mainardi [1] proposed a modified fractional differentiation operator $D_{t}^{\alpha }$ to describe the theory of viscoelasticity in order to overcome the discrepancy of the Riemann–Liouville derivative [4,11]. It is mentioned that the proposed Caputo fractional derivative allows the utilization of initial and boundary conditions involving integer order derivatives.

**Definition 3.3 [1,11]**

The fractional derivative of $f\in C_{\mu }$ of the order *α*≥0, in Caputo sense, is defined as

3.2 $$D_{t}^{\alpha }f(t)=J_{t}^{m-\alpha }D_{t}^{m}f(t)=\frac{1}{\Gamma (m-\alpha )}\int_{0}^{t}{\left(t-\tau \right)}^{m-\alpha -1}f^{\left(m\right)}(\tau )\;d\tau ,$$

for $m-1<\alpha \leq m,\;m\in N,\;t>0,\;f\in C_{\mu }^{m}$, *μ*≥−1.

The basic properties of Caputo fractional derivative are given as follows:

**Lemma 3.4 [1–3,11]**

*Let m*−1<*α*≤*m*, m∈N, *and* $f\in C_{\mu }^{m},$ *μ*≥−1, *then*

DtαJtαf(t)=f(t)JtαDtαf(t)=f(t)−∑k=0mf(k)(0+)tkk!,for t>0.

In this work, the Caputo fractional derivative is considered because it includes traditional initial and boundary conditions in the formulation of the physical problems. For more details on fractional derivatives, one can refer to [2–11].

### 3.1 Fractional reduced differential transform method

In this section, basic properties of FRDTM are described [57–59]. Let *ψ*(*x*,*t*) be a function of two variables such that *ψ*(*x*,*t*)=*f*(*x*)*g*(*t*), then from the properties of the one-dimensional differential transform (DT) method, we have

3.3 $$\psi (x,t)=\sum_{i=0}^{\infty }f(i)x^{i}\sum_{j=0}^{\infty }g(j)t^{j}=\sum_{i=0}^{\infty }\sum_{j=0}^{\infty }\Psi (i,j)x^{i}t^{j},$$

where *ψ*(*i*,*j*)=*f*(*i*)*g*(*j*) is referred to as the spectrum of *ψ*(*x*,*t*). Throughout the paper, *R*_D_ and *R*^−1^_D_ denote the operators for fractional reduced differential transform (FRDT) and inverse FRDT, respectively. Furthermore, the lowercase *ψ*(*x*,*t*) is used for the original function, whereas its fractional reduced transformed function is represented by the uppercase *Ψ*_*k*_(*x*).

The basic definitions and properties of FRDTM are described below.

**Lemma 3.5 [57,59]**

*Let ψ*(*x*,*t*) *be an analytic and continuously differentiable with respect to space variable x and time variable t in the domain of interest, then*

(*a*) *FRDT of ψ is given by*
  $$\Psi_{k}(x)=\frac{1}{\Gamma (k\alpha +1)}[D_{t}^{k}(\psi (x,t)){]}_{t=t_{0}},\;k=0,1,2,\ldots$$
  *where α describes the order of time fractional derivative*.
(*b*) *The inverse FRDT of **Ψ***_*k*_(*x*) *is defined by*
  $$\psi (x,t):=\sum_{k=0}^{\infty }\Psi_{k}(x){\left(t-t_{0}\right)}^{k\alpha }.$$
(*c*) *From* (a) *and* (b), *we have*
  $$\psi (x,t)=\sum_{k=0}^{\infty }\frac{1}{\Gamma (k\alpha +1)}[D_{t}^{k}(\psi (x,t)){]}_{t=t_{0}}{\left(t-t_{0}\right)}^{k\alpha }.$$
  *In particular, for t*_0_=0, *the above equation becomes*
  $$\psi (x,t)=\sum_{k=0}^{\infty }\frac{1}{\Gamma (k\alpha +1)}[D_{t}^{k}(\psi (x,t)){]}_{t=0}t^{k\alpha }.$$

This shows that FRDTM is a special case of the power series expansion.

**Lemma 3.6 [57–60]**

*Let u*(*x*,*t*) *and v*(*x*,*t*) *be any two analytic and continuously differentiable functions with respect to space variable x and time t such that u*(*x*,*t*)=*R*^−1^_D_[*U*_*k*_(*x*)] *and v*(*x*,*t*)=*R*^−1^_D_[*V*_*k*_(*x*)], *then*

$$\;\left(a\right)\;R_{D}\{u(x,t)v(x,t)\}=U_{k}(x)⊗V_{k}(x)=\sum_{r=0}^{k}U_{r}(x)V_{k-r}(x);$$

$$\;\left(b\right)\;R_{D}\{a_{1}u(x,t)\pm a_{2}v(x,t)\}=a_{1}U_{k}(x)\pm a_{2}V_{k}(x);$$

(c)RD{xmtnu(x,t)}=xmUk−n(x)if k≥n0,else.

$$\;\left(d\right)\;R_{D}\{D_{t}^{N\alpha }(u(x,t))\}=\frac{\Gamma (1+(k+N)\alpha )}{\Gamma (1+k\alpha )}U_{k+N}(x);$$

$$\;\left(e\right)\;R_{D}\{D_{x}^{l}u(x,t)\}=D_{x}^{l}U_{k}(x);\;R_{D}\{x^{m}\}=x^{m}\delta (k)\;and\;R_{D}\{e^{\lambda t}\}=\frac{\lambda^{k}}{k!},$$

*where the convolution* ⊗ *denotes the fractional reduced differential transform version of multiplication and the function δ is defined by*

δ(k):=1if k=00otherwise.

## 4. Numerical results and discussion

In this section, four examples of time fractional-order multi-dimensional diffusion equations are considered to validate the reliability and efficiency of FRDTM. The approximate analytical solutions are obtained by considering forty grid points and the first 20 terms of the series.

**Example 4.1**

Consider the one-dimensional time fractional-order heat-like diffusion equation [44]

4.1 $$D_{t}^{\alpha }u=\frac{x^{2}}{2}\;\frac{\partial^{2}u}{\partial x^{2}},\;\forall \;x\in [0,1],\;t>0,\;0<\alpha \leq 1,$$

subject to initial concentration

4.2 $$u(x,0)=x^{2}.$$

The following recurrence relation is obtained by applying FRDTM to equation (4.1):

4.3 $$\frac{\Gamma \left((1+k)\alpha +1\right)}{\Gamma \left(1+k\alpha \right)}U_{k+1}(x)=\frac{x^{2}}{2}\frac{\partial^{2}U_{k}(x)}{\partial x^{2}}.$$

Now, using FRDTM to the initial condition (4.2), we obtain

4.4 $$U_{0}(x)=x^{2}.$$

Using the above equation in equation (4.3), the following recursive values of *U*_*k*_ are obtained successively:

4.5 $$U_{1}(x)=\frac{x^{2}}{\Gamma \left(1+\alpha \right)};\;U_{2}(x)=\frac{x^{2}}{\Gamma \left(1+2\alpha \right)};\;U_{3}(x)=\frac{x^{2}}{\Gamma \left(1+3\alpha \right)};\;\ldots ;\;U_{k}(x)=\frac{x^{2}}{\Gamma \left(1+k\alpha \right)};\;\ldots$$

Next, using the inverse FRDT of *U*_*k*_(*x*) and equation (4.5), we have

4.6 $$\begin{array}{rl} u(x,t) & =\sum_{k=0}^{\infty }U_{k}(x)t^{\alpha k}=\sum_{k=0}^{\infty }\frac{x^{2}}{\Gamma \left(1+k\alpha \right)}t^{k\alpha }=x^{2}\sum_{k=0}^{\infty }\frac{t^{k\alpha }}{\Gamma \left(1+k\alpha \right)}=x^{2}E_{\alpha }(t^{\alpha }), \end{array}$$

where $E_{\alpha }(t^{\alpha })=\sum_{k=0}^{\infty }(t^{k\alpha }/\Gamma \left(1+k\alpha \right))$ is the Mittag–Leffler function. Equation (4.6) represents the exact solution of (4.1). The same solution was obtained by Momani [44] using ADM. In particular, when α→1, equation (4.6) reduces to

**Figure 1.**
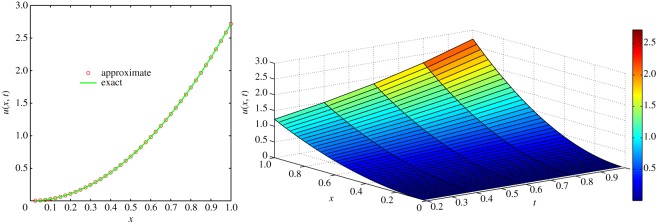
Comparison of exact concentration with approximate concentration (left), and physical behaviour (right) of one-dimensional classical heat-like diffusion equation (4.1) at *t*=1.

**Figure 2.**
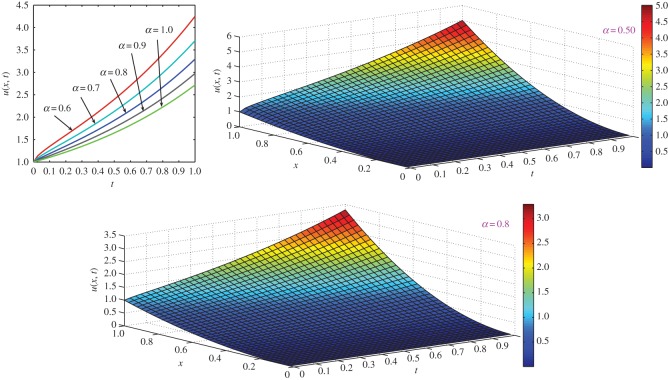
Approximate concentration of one-dimensional fractional heat-like diffusion equation (4.1) for different values of *α*≤1 and *t*≤1.

4.7 $$u(x,t)=x^{2}\sum_{k=0}^{\infty }\frac{t^{k}}{\Gamma \left(1+k\right)}=x^{2}\;e^{t},$$

which is the exact solution of the one-dimensional classical heat-like diffusion equation (i.e. equation (4.1) with *α*=1). The above result is in complete agreement with the result obtained by Momani [44]. For *α*=1, comparison of exact concentration with approximate concentration at *t*=1 and the physical behaviour is depicted in figure 1. The approximate behaviour of concentration for different values of *α*≤1 is depicted in figure 2.

**Example 4.2**

Consider the two-dimensional time fractional-order diffusion equation [55]

4.8 $$D_{t}^{\alpha }u=\frac{\partial^{2}u}{\partial x^{2}}+\frac{\partial^{2}u}{\partial y^{2}},\;\forall \;x,y\in [0,1],\;t>0\;0<\alpha \leq 1$$

subject to initial concentration which grows exponentially in *x* and *y* as follows:

4.9 $$u(x,y,0)=e^{x+y}.$$

The following recurrence relation is obtained by applying FRDTM to equation (4.8):

4.10 $$\frac{\Gamma \left((1+k)\alpha +1\right)}{\Gamma \left(1+k\alpha \right)}U_{k+1}(x,y)=\frac{\partial^{2}U_{k}(x,y)}{\partial x^{2}}+\frac{\partial^{2}U_{k}(x,y)}{\partial y^{2}}.$$

Now, using FRDTM to the initial condition (4.9), we have

4.11 $$U_{0}(x,y)=e^{x+y}.$$

Using the above equation in equation (4.10), the following recursive values of *U*_*k*_ are obtained successively:

4.12 $$\left.\begin{array}{rl} U_{1}(x,y) & =\frac{2}{\Gamma \left(1+\alpha \right)}\;e^{x+y};\;U_{2}(x,y)=\frac{2^{2}}{\Gamma \left(1+2\alpha \right)}\;e^{x+y};\\ \;U_{3}(x,y) & =\frac{2^{3}}{\Gamma \left(1+3\alpha \right)}\;e^{x+y};\;\ldots ;\;U_{k}(x,y)=\frac{2^{k}}{\Gamma \left(1+k\alpha \right)}\;e^{x+y};\;\ldots \end{array}\right\}$$

Next, using the inverse FRDT of *U*_*k*_(*x*,*y*) and equation (4.12), we have

4.13 $$\begin{array}{rl} u(x,y,t) & =\sum_{k=0}^{\infty }U_{k}(x,y)t^{\alpha k}=U_{0}(x,y)+U_{1}(x,y)t^{\alpha }+U_{2}(x,y)t^{2\alpha }+\cdots +U_{k}(x,y)t^{\alpha k}+\cdots \\ & =e^{x+y}\left\{1+\frac{2t^{\alpha }}{\Gamma \left(1+\alpha \right)}+\frac{2^{2}t^{2\alpha }}{\Gamma \left(1+2\alpha \right)}+\frac{2^{3}t^{3\alpha }}{\Gamma \left(1+3\alpha \right)}+\cdots +\frac{2^{k}t^{k\alpha }}{\Gamma \left(1+k\alpha \right)}+\cdots \right\}\\ & =e^{x+y}\sum_{k=0}^{\infty }\frac{{\left(2t^{\alpha }\right)}^{k}}{\Gamma \left(1+k\alpha \right)}=e^{x+y}E_{\alpha }(2t^{\alpha }), \end{array}$$

which is the exact solution of equation (4.8). Furthermore, when α→1, equation (4.13) reduces to

**Figure 3.**
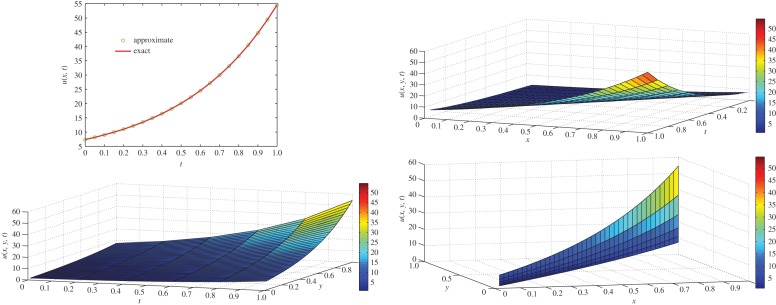
Comparison of exact concentration with approximate concentration (top left), and the behaviour of concentration of two-dimensional diffusion equation (4.8) with respect to different axes for *α*=1 at *t*=1.

**Figure 4.**
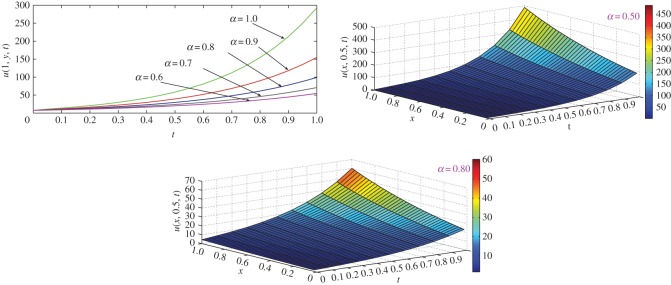
Approximate concentration of two-dimensional fractional diffusion equation (4.8) for different values of *α*≤1 at different time levels *t*≤1.

4.14 $$u(x,y,t)=e^{x+y}\sum_{k=0}^{\infty }\frac{{\left(2t\right)}^{k}}{\Gamma \left(1+k\right)}=e^{x+y+2t},$$

which is the exact solution for the two-dimensional classical diffusion equation (i.e. equation (4.8) with *α*=1). The same solution was obtained by Kumar *et al.* [55] using a modified homotopy perturbation method. For *α*=1 and *t*=1, comparison of exact concentration with approximate concentration as well as the behaviour of concentration of the two-dimensional classical diffusion equation (4.8) with respect to different axes is depicted in figure 3. Moreover, the behaviour of concentration of fractional diffusion equation (4.8) for different values of *α*≤1 is depicted in figure 4.

**Example 4.3**

Consider the three-dimensional time fractional-order diffusion equation [55]

4.15 $$D_{t}^{\alpha }u=\frac{\partial^{2}u}{\partial x^{2}}+\frac{\partial^{2}u}{\partial y^{2}}+\frac{\partial^{2}u}{\partial z^{2}},\;\forall \;x,y,z\in [0,1],\;t>0,\;0<\alpha \leq 1,$$

subject to initial concentration which grows exponentially in both *x* and *z* as follows:

4.16 $$u(x,y,z,0)=(1-y)\;e^{x+z}.$$

The following recurrence relation is obtained by applying FRDTM to equation (4.15):

4.17 $$\frac{\Gamma \left((1+k)\alpha +1\right)}{\Gamma \left(1+k\alpha \right)}U_{k+1}(x,y,z)=\frac{\partial^{2}U_{k}(x,y,z)}{\partial x^{2}}+\frac{\partial^{2}U_{k}(x,y,z)}{\partial y^{2}}+\frac{\partial^{2}U_{k}(x,y,z)}{\partial z^{2}}.$$

Now, using FRDTM to the initial condition (4.16), we have

4.18 $$U_{0}(x,y,z)=(1-y)\;e^{x+z}.$$

Using the above equation in equation (4.17), the following recursive values of *U*_*k*_ are obtained successively:

4.19 $$\left.\begin{array}{rl} U_{1}(x,y,z) & =\frac{2}{\Gamma \left(1+\alpha \right)}(1-y)\;e^{x+z};\;U_{2}(x,y,z)=\frac{2^{2}}{\Gamma \left(1+2\alpha \right)}(1-y)\;e^{x+z};\\ \;U_{3}(x,y,z) & =\frac{2^{3}}{\Gamma \left(1+3\alpha \right)}(1-y)\;e^{x+z};\;\ldots ;\;U_{k}(x,y,z)=\frac{2^{k}}{\Gamma \left(1+k\alpha \right)}(1-y)\;e^{x+z};\;\ldots \end{array}\right\}$$

Next, using the inverse FRDT of *U*_*k*_(*x*,*y*,*z*) and equation (4.19), we have

4.20 $$\begin{array}{rl} u(x,y,z,t) & =U_{0}(x,y,z)+U_{1}(x,y,z)t^{\alpha }+U_{2}(x,y,z)t^{2\alpha }+\cdots +U_{k}(x,y,z)t^{\alpha k}+\cdots \\ & =(1-y)\;e^{x+z}\left\{1+\frac{2t^{\alpha }}{\Gamma \left(1+\alpha \right)}+\frac{2^{2}t^{2\alpha }}{\Gamma \left(1+2\alpha \right)}+\frac{2^{3}t^{3\alpha }}{\Gamma \left(1+3\alpha \right)}+\cdots +\frac{2^{k}t^{k\alpha }}{\Gamma \left(1+k\alpha \right)}+\cdots \right\}\\ & =(1-y)\;e^{x+z}\sum_{k=0}^{\infty }\frac{{\left(2t^{\alpha }\right)}^{k}}{\Gamma \left(1+k\alpha \right)}=(1-y)\;e^{x+z}E_{\alpha }(2t^{\alpha }), \end{array}$$

which is the exact solution of (4.15). For α→1, equation (4.20) reduces to

**Figure 5.**
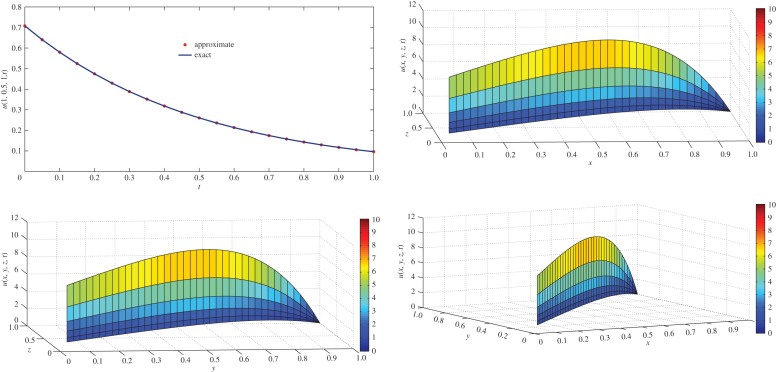
Comparison of exact concentration with approximate concentration (top left) at *t*=1, and the behaviour of concentration of three-dimensional classical diffusion equation (4.15) with respect to different axis at *t*=1.

4.21 $$u(x,y,z,t)=(1-y)\;e^{x+z}\sum_{k=0}^{\infty }\frac{{\left(2t\right)}^{k}}{\Gamma \left(1+k\right)}=(1-y)\;e^{x+z+2t},$$

which is the exact solution for the three-dimensional classical diffusion equation. The same solution was obtained by Kumar *et al.* [55] using a modified homotopy perturbation method. For *α*=1 and *t*=1, comparison of exact concentration with approximate concentration as well as the behaviour of concentration of the two-dimensional diffusion equation (4.15) with respect to different axes is depicted in figure 5.

**Example 4.4**

Consider the three-dimensional time fractional-order inhomogeneous heat-like diffusion equation [44]

4.22 $$D_{t}^{\alpha }u=x^{4}y^{4}z^{4}+\frac{1}{36}\left(x^{2}\;\frac{\partial^{2}u}{\partial x^{2}}+y^{2}\;\frac{\partial^{2}u}{\partial y^{2}}+z^{2}\;\frac{\partial^{2}u}{\partial z^{2}}\right),\;\forall \;x,y,z\in [0,1],\;t>0,\;0<\alpha \leq 1,$$

subject to ‘zero’ initial concentration as

4.23 u(x,y,z,0)=0.

The following recurrence relation is obtained by applying FRDTM to equation (4.22):

4.24 $$\begin{array}{r} \frac{\Gamma \left((1+k)\alpha +1\right)}{\Gamma \left(1+k\alpha \right)}U_{k+1}(x,y,z)=x^{4}y^{4}z^{4}\delta (k)+\frac{1}{36}\left(x^{2}\frac{\partial^{2}U_{k}(x,y,z)}{\partial x^{2}}+y^{2}\frac{\partial^{2}U_{k}(x,y,z)}{\partial y^{2}}+z^{2}\frac{\partial^{2}U_{k}(x,y,z)}{\partial z^{2}}\right). \end{array}$$

Now, using FRDTM to the initial condition (4.23), we have

4.25 $$U_{0}(x,y,z)=0.$$

Using the above equation into equation (4.24), the following recursive values of *U*_*k*_ are obtained successively:

4.26 $$\left.\begin{array}{rl} U_{1}(x,y,z) & =\frac{x^{4}y^{4}z^{4}}{\Gamma \left(1+\alpha \right)};\;U_{2}(x,y)=\frac{x^{4}y^{4}z^{4}}{\Gamma \left(1+2\alpha \right)};\\ \;U_{3}(x,y) & =\frac{x^{4}y^{4}z^{4}}{\Gamma \left(1+3\alpha \right)};\;\ldots ;\;U_{k}(x,y)=\frac{x^{4}y^{4}z^{4}}{\Gamma \left(1+k\alpha \right)};\;\ldots \end{array}\right\}$$

Next, using the inverse FRDT of *U*_*k*_(*x*,*y*,*z*) and equation (4.26), we have

4.27 $$\begin{array}{rl} u(x,y,z,t) & =U_{0}(x,y,z)+U_{1}(x,y,z)t^{\alpha }+U_{2}(x,y,z)t^{2\alpha }+U_{3}(x,y,z)t^{3\alpha }\\ & \;+\cdots +U_{k}(x,y,z)t^{\alpha k}+\cdots \\ & =x^{4}y^{4}z^{4}\left\{\frac{t^{\alpha }}{\Gamma \left(1+\alpha \right)}+\frac{t^{2\alpha }}{\Gamma \left(1+2\alpha \right)}+\frac{t^{3\alpha }}{\Gamma \left(1+3\alpha \right)}+\cdots +\frac{t^{k\alpha }}{\Gamma \left(1+k\alpha \right)}+\cdots \right\}\\ & =x^{4}y^{4}z^{4}\;\left(\sum_{k=0}^{\infty }\frac{{\left(t^{\alpha }\right)}^{k}}{\Gamma \left(1+k\alpha \right)}-1\right)=x^{4}y^{4}z^{4}(E_{\alpha }(t^{\alpha })-1), \end{array}$$

which is the exact solution of (4.22). For α→1, equation (4.27) reduces to

**Figure 6.**
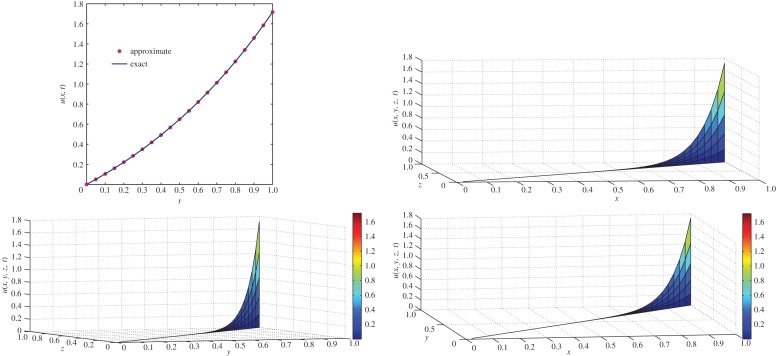
Comparison of exact concentration with approximate concentration (top left) for *α*=1, and physical behaviour of classical heat-like diffusion equation (4.22) with respect to different axes at *t*=1.

**Figure 7.**
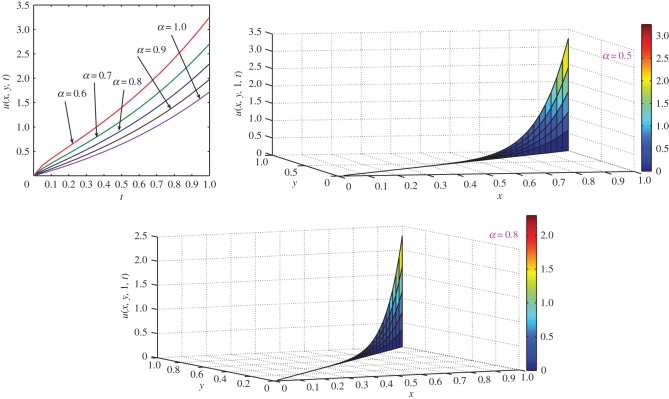
Approximate concentration of three-dimensional heat-like diffusion equation (4.22) for different values of *α*≤1 at different time levels *t*≤1.

4.28 $$u(x,y,z,t)=x^{4}y^{4}z^{4}\sum_{k=1}^{\infty }\frac{{\left(t\right)}^{k}}{\Gamma \left(1+k\right)}=x^{4}y^{4}z^{4}(e^{t}-1),$$

which is the exact solution of the three-dimensional classical heat-like diffusion equation. The same solution was obtained by Momani [44] using ADM. For *α*=1, comparison of exact concentration with approximate concentration at *t*=1 and physical behaviour with respect to different axes are depicted in figure 6. The approximate behaviour of concentration for different values of *α*≤1 are depicted in figure 7.

## 5. Conclusion

In this study, FRDTM is implemented successfully to find out the analytical solution of the time fractional-order multi-dimensional diffusion equation in terms of an infinite power series for the appropriate initial condition. The proposed approximate solutions are obtained without any transformation, perturbation, discretization or any other restrictive conditions. Four examples are carried out to study the accurateness and effectiveness of the technique. The proposed solutions by FRDTM are in excellent agreement with those obtained Kumar *et al.* [55] using M-HPM, and Momani [44] using ADM. The small size of calculation in FRDTM in comparison with M-HPM is its main advantage.

## Data accessibility

The manuscript has no supporting data.
